# Prediction of preterm birth at St. Mary's Hospital Lacor, Northern Uganda: a prospective cohort study

**DOI:** 10.4314/ahs.v24i2.31

**Published:** 2024-06

**Authors:** Silvia Awor, Rosemary Byanyima, Benard Abola, Annettee Nakimuli, Christopher Orach, Paul Kiondo, Jasper Ogwal Okeng, Dan Kaye

**Affiliations:** 1 Gulu University, Reproductive Health; 2 Mulago National Referral Hospital, Radiology; 3 Gulu University, Mathematics; 4 Makerere University, Obstetrics and Gynaecology; 5 Makerere University CHS, School of Public Health; Community Health; 6 Lira University, Pharmacology

**Keywords:** Prediction, second-trimester, preterm-birth, Uganda, Africa

## Abstract

**Background:**

Preterm birth causes over 2% of perinatal mortality in Africa. Screening in prenatal clinics, may be used to identify women at risk. This study developed and validated second-trimester prediction models of preterm birth, using maternal socio-demographic characteristics, sonographic findings, and laboratory parameters in Northern Uganda.

**Methods:**

This prospective cohort study recruited 1,000 pregnant mothers at 16 - 24 weeks, and assessed their socio-demographic and clinical characteristics. Preterm birth (delivery after 28 and before 37 weeks) was the primary study outcome. Multi-variable analyses were performed, built models in RStudio, and cross-vaidated them using K (10)-fold cross-validation.

**Results:**

The Incidence of preterm birth was 11.9% (90 out of 774). The predictors of preterm birth were multiple pregnancies, personal history of preeclampsia, history of previous preterm birth, diastolic hypertension, serum ALP<98IU, white blood cell count >11000 cells/µl, platelet lymphocyte ratio >71.38, serum urea of 11-45 IU. These predicted preterm birth by 69.5% AUC, with 62.4% accuracy, 77.2% sensitivity, and 47.1% specificity.

**Conclusion:**

Despite low specificity, these models predict up to 77.2% of those destined to have a preterm birth, and may be used for second-trimester preterm birth screening in low-resource clinics.

## Introduction

Preterm birth is the termination of pregnancy before 37 completed weeks of gestation^1^. In Uganda, and other low-resource settings, preterm birth is the termination of pregnancy between 28 and 37 weeks^2^. Preterm birth contributes to over 2% of perinatal morbidity and mortality^3^, ^4^, and management requires a multidisciplinary team, and specialized equipment^5^-^8^. Nonetheless, African ancestry is considered a risk factor for preterm birth^9^.

Prediction of preterm birth using maternal social demographic features has been attempted. For example, in South Korea, maternal age, maternal body mass index (BMI), prior preterm birth, education, occupation, income, and active and passive smoking predicted preterm birth by >90%^10^. In Brazil, smoking and a history of previous preterm birth <34 weeks predicted preterm birth by 67% area under the curve (AUC)^11^.

Ultrasonography and uterine artery doppler flow indices have also been done in some communities. In Spain and Sweden, a short cervical length on ultrasound of endo-cervical length of ≤25 mm, predicted preterm birth at <33 weeks of gestation, with 38.5% sensitivity and 95.8% specificity, and an area under the curve (AUC) of about 64%^12^, ^13^. When the cervicovaginal thrombospondin-1 level was used in Germany, an AUC of 86% with 94% sensitivity, and 77% specificity was obtained for predicting preterm birth^14^.

Laboratory tests have also been done, to predict preterm birth. For example, in Cuba, placental alpha microglobulin-1 (PAMG-1) had 100% sensitivity and 11% specificity^15^. In the USA, twenty-five serum biomarkers, maternal age<34 years, and poverty status identified >80% of women with preterm birth^16^.

Overall, Meertens et al.^9^ found, in a systemic review, that most models had an AUC of 54%-70% for both the development and validation of prediction models for preterm birth. The studies included in these reviews were primarily conducted in high-income settings and outside Africa, despite the higher burden of preterm birth in low-resource settings.

There is limited data within Africa, and Uganda, for predicting preterm birth. Thus, we investigated the predictors of preterm birth, and developed and validated second-trimester prediction models, using maternal socio-demographic characteristics, sonographic findings, and laboratory parameters in Northern Uganda.

## Methods

**Study design and setting:** This was a prospective cohort study at St. Mary's Hospital Lacor. It is a private, not-for-profit hospital, founded by the Catholic Church. It is located six kilometres West of Gulu city, along Juba Road in Gulu District (Longitude 30 – 32 degrees East and Latitude 02 – 04 degrees North). St. Mary's Hospital Lacor is one of the teaching hospitals of Gulu University, with a bed capacity of 482. The hospital receives over nine thousand antenatal mothers, and conducts about 7,000 deliveries per year^17^. This study site was chosen, because the population is understudied, and it was convenient for some authors, during data collection.

### Study population

The target population was all pregnant women attending prenatal care at St. Mary's hospital Lacor. The accessible population came to prenatal care during the study period. We recruited 1,000 pregnant mothers, 16 – 24 weeks, from April 2019 to March 2020, and gave them unique identifiers (study numbers). All participants with lethal congenital anomalies were excluded.

### Sample size estimation

Using Yamane's 1967 formula^18^ for calculating sample size for cohort studies, using finite population size, St. Mary's hospital Lacor delivers approximately 7,000 babies per year. Since my study duration is 12 months for recruitment of the mothers, the finite population I can access is about 7,000 mothers.

Yamane 1967 formula: Sample size n = N / 1+Ne^2^

Where N is the finite population size of 7,000 mothers

The margin of error (e) 5%

Therefore, n = 7,000 / 1+7,000(0.05)^2^

n = 379

The required sample size was 379 mothers. We doubled the number (to >758), to cater for loss to follow-up, miscarriages, and clients opting out of the study during the follow-up period, since the rate of hospital delivery in this region is low (about 55% only^19^). In addition, we tripled the sample size, because we expected some people to deliver from the free government hospitals nearby.

Another sample size calculation was done using an online tool, giving a sample size of 580 mothers^20^. Again, we assumed an infinite population with 80% power, 95% confidence level, 10 minimum number of predictors, 5% margin of error, and 10% rate of occurrence^20^. Also doubled the sample size, to compensate for the loss to follow-up.

### Data collection

Started recruitment of participants from 3^rd^ April 2019, till 26^th^ March 2020. Six midwives working in the prenatal clinic were all trained for an hour, to obtain informed consent for the study, fill out the questionnaire, take blood and urine samples of the participants, direct the participants for Doppler ultrasound, and hand over the samples to the laboratory technicians. A consecutive sampling of the mothers who satisfied the inclusion criteria was done. A questionnaire was filled out with the help of a midwife (research assistant). Blood samples were taken for complete blood count (using Huma-Count 80Ts made in Germany), liver and renal function tests (using Humalyzer 2000 made in Germany), and uterine artery Doppler sonography (using GE Loqig V2 ultrasound). All results were measured in the known standard units. Ratios and indices like platelet-lymphocyte ratio, pulsatility and resistive indices had no units. The laboratory technicians and sonographers only used the study numbers to identify the participants. The mothers were followed up until 30^th^ September 2020, when the last one was delivered. Therefore, the delivery team had no access to the questionnaire, laboratory and ultrasound results.

### Study variables

*Predictor variables:* Social demographic characteristics, maternal history and physical examination findings were recorded. From social demographic characteristics and maternal history, grouping was based on known classifixation, e.g. level of education in Uganda is grouped as primary, secondary and tertiary education levels. In addition, gravidity was grouped as prime gravida for first pregnancies, multigravida for 2-4 and grand multigravida >4.

The laboratory tests were grouped according to the reference ranges given by the laboratory. While ultrasound pulsatility and resistive indices were grouped using percentiles. The end-diastolic notch of the uterine arteries was either present or absent; if present, it was either unilateral or bilateral. The placental location and length of the cervix were also recorded. The participants with incomplete results on any of the main study variables were dropped from the final analysis

### Outcome variable

Delivery of the foetus from 28 weeks to <37 weeks was taken as preterm birth. The mothers who delivered before 28 weeks were excluded from the analysis.

### Statistical analysis

Data were pre-processed using Stata® 15.0 and built models in RStudio (R version 4.1.1 (2021-08-10). Seven hundred seventy-four (774) complete delivery records were obtained for mothers who delivered at ≥28 weeks. Used proportions to compute the incidence of preterm birth.

Bivariable analysis was done, and all variables with p-values ≤0.20 or were known risk factors for preterm birth, like maternal age and maternal comorbidities, were included in a logistic regression model. The statistically significant predictors of preterm birth in the logistic regression model with p<0.05, were taken as independent risk factors for preterm birth.

For a binary outcome like term and preterm births, it needs them to be near 50% proportion to obtain a more accurate prediction model in RStudio^21^, ^22^. Since there were few preterm births compared to term births, over-sampling of preterm births and under-sampling of term births (the ROSE technique)^21^, ^22^ balanced the dataset, and obtained a distribution of term and preterm births, as 394 (50.9%) and 380 (49.1%), respectively. The ROSE-derived data set was fitted into a confusion matrix, to evaluate the performance of our models' area, under the curve (AUC) with accuracy, sensitivity and specificity, using K-(10)-fold cross-validation.

### Ethical consideration

The study was approved by the Research and Ethics Committee of Makerere University School of Medicine (reference number 2018-105), Uganda National Council for Science and Technology (Reference number HS258ES), and administrative clearance from St. Mary's Hospital Lacor (Reference number LHIREC Adm 009/11/18). Written informed consent was sought from every participant. All records are kept under a safe lock, by the principal investigator.

## Results

**Figure 1:**
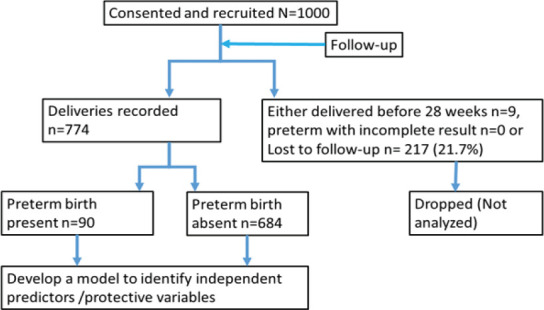
Showing the flow of participants through the study for preterm birth prediction

One thousand (1,000) participants were recruited, and followed up. By the end of the study period, seven hundred eighty-three (783) delivery records were complete, while 21.7% (217 out of 1,000) participants were lost to follow-up. Nine out of the 783 were born before 28 weeks (miscarriage), and were excluded from the final analysis. Some follow-up periods coincided with covid-19 lockdowns (Figure 1).

About 12.5% (99 out of 783) of the *foeti* were born before 37 completed weeks of gestation, with 1.0% (9 out of 783) occurring at <28 weeks, and 11.6% (90 out of 774) at 28-<37 weeks. However, by Uganda's definition of 28 weeks being the age of viability, the incidence of preterm birth was 11.6%.

### Baseline characteristics of mothers who had preterm and term births

Those who delivered preterm were more likely to have a history of preeclampsia, preterm birth, diastolic hypertension, end-diastolic notch, leukocytosis or multiple pregnancies. (Table 1)

**Table 1:** Baseline characteristics of mothers who had preterm and term births

Characteristics (n=774)	Term births n=684	Preterm n=90	P_value
**Personal history of preeclampsia**	4 (40.0%)	6 (60.0%)	0.000
**Not applicable (prime gravida) (normal)**	216 (88.5%)	28 (11.5%)	
**No personal history of preeclampsia (normal)**	464 (89.2%)	56 (10.8%)	
**History of preterm birth**	56 (77.8%)	16 (22.2%)	0.003
**Not applicable (prime gravida) (normal)**	216 (88.5%)	28 (11.5%)	
**No history of preterm birth (normal)**	412 (90.0%)	46 (10.0%)	
**Multiple pregnancies**	7 (41.2%)	10 (58.8%)	0.000
**Singleton pregnancy (normal)**	677 (89.4%)	80 (10.6%)	
**Diastolic blood pressure <90mmHg (normal)**	668 (88.9%)	83 (11.1%)	0.004
**Diastolic blood pressure ≥90mmHg**	16 (69.6%)	7 (30.4%)	
**No end diastolic notch (normal)**	498 (89.0%)	62 (11.0%)	0.037
**Unilateral notch**	116 (91.3%)	11 (8.7%)	
**Bilateral notch**	70 (80.5%)	17 (19.5%)	
**MCV <80 fl**	182 (91.0%)	18 (9.0%)	0.351
**MCV 80 - 100 fl (normal)**	490 (87.4%)	71 (12.6%)	
**MCV ≥100 fl**	12 (92.3%)	1 (7.7%)	
**White blood cell count <4000 cells/µl**	76 (84.4%)	14 (15.6%)	0.022
**White cell count of 4000 - 11000 cells/µl (normal)**	597 (89.1%)	71 (10.9%)	
**White cell count of > 11000cells/µl**	11 (68.8%)	4 (31.2%)	
**Lymphocyte count <900 cells/µl**	38 (77.6%)	11 (22.4%)	0.019
**Lymphocyte count 900 - 3900 cells/µl (normal)**	632 (89.2%)	76 (10.8%)	
**Lymphocyte count >3900 cells/µl**	14 (77.8%)	2 (22.2%)	
**Platelet count <150 cells/µl**	83 (90.2%)	9 (8.8%)	0.377
**Platelet count 150 – 400 cells/µl (normal)**	591 (88.2%)	78 (11.8)	
**Platelet count >400 cells/µl**	10 (76.9%)	3 (23.1%)	
**Serum ALP < 98 IU/L**	22 (78.6%)	6 (21.4%)	0.106
**Serum ALP ≥ 98 IU/L (normal but unreliable (23))**	662 (88.6%)	84 (11.4%)	
**PLR <71.38 (First quadrant)**	70 (94.6%)	4 (5.4%)	0.204
**PLR of 71.38 - 212.3 (Second and third quadrants)**	544 (87.6%)	76 (12.4%)	
**PLR of > 212.3 (Fourth Quadrant)**	70 (87.5%)	10 (12.5%)	
**AST <4 IU/L**	66 (94.4%)	4 (5.6%)	0.080
**Serum AST of 4 - 40 IU/L (normal)**	550 (87.2%)	81 (12.8%)	
**Serum AST of >40 IU/L**	68 (93.1%)	5 (8.9%)	
**Serum sodium <135.1 mEq/dL**	163 (85.3%)	28 (14.7%)	0.280
**Serum sodium of 135.1 - 139.4 mEq/dL (normal)**	361 (88.9%)	45 (11.1%)	
**Serum sodium of >139.4 mEq/dL**	160 (90.4%)	17 (9.6%)	
**Serum Urea >45 IU/L**	74 (94.8%)	4 (5.2%)	0.157
**Serum urea of 11 - 45 IU/L (normal)**	564 (87.6%)	80 (12.4%)	
**Serum urea of <11 IU/L**	46 (88.5%)	6 (11.5%)	

IU/L=international units per litre, mEq/dL=milliequivalent per deciliter, cells/µl=cells pre microlitre, fl=femtoliter, mmHg=millimeters of mercury.

### Unadjusted Prediction for preterm birth

History of previous preterm birth, personal history of preeclampsia, multiple pregnancy, and diastolic hypertension, was predictive of preterm birth (Table 2).

**Table 2:** Bivariable analysis of predictors of preterm birth

Variable n=774	Preterm / Term	OR (95% CI)	p-value
Maternal history, physical findings			

Nulliparity	28/217	1.43 (0.86 - 2.40)	0.173
Para 1-2	43/313	1.52 (0.94 - 2.45)	0.090
History of previous preterm birth	16/56	2.07 (1.29 - 3.32)	0.003
History of abortion	23/130	1.37 (0.92 - 2.01)	0.116
Personal history of preeclampsia	6/4	4.72 (2.77 - 8.04)	0.000
Alcohol use in pregnancy	3/45	0.44 (0.15 - 1.35)	0.151
Multiple pregnancies	10/7	4.96 (3.25 - 7.56)	0.000
Diastolic pressure ≥ 90mmHg	7/16	2.53 (1.32 - 4.83)	0.005

Maternal sonographic findings			

Average pulsatility index >1.19 (>90th percentile)	11/71	1.58 (1.01 - 2.47)	0.046
Average resistive index >0.65 (>90th percentile)	11/74	1.45 (0.91 - 2.30)	0.115
Unilateral end diastolic notch	11/116	1.16 (0.72 - 1.86)	0.536
Bilateral end diastolic notch	17/70	1.96 (1.29 - 2.99)	0.002
Cervical length <25 mm	1/15	0.53 (0.07 - 3.60)	0.518

Maternal laboratory tests			

Serum albumin 3.5 - 4.1 mg/dL (25th - 75th percentile)	44/366	0.71 (0.45 - 1.11)	0.134
Serum albumin >4.1 mg/dL (>75th percentile)	22/169	0.80 (0.47 - 1.35)	0.399
Serum AST of 4 - 40 IU/L (10th - 90th percentile)	81/554	2.27 (0.86 - 5.99)	0.100
Serum AST of >40 IU/L (>90th percentile)	5/65	1.20 (0.34 - 4.29)	0.780
Serum urea of 11 - 45 IU/L (10th - 90th percentile)	80/572	2.44 (0.92 - 6.49)	0.073
Serum urea of <11 IU/L (<10th percentile)	6/46	2.25 (0.67 - 7.59)	0.191
Serum ALP < 98 IU/L	6/22	1.88 (0.90 - 3.93)	0.093
Lymphocyte count 900 - 3900 cells/µl	77/634	0.48 (0.27 - 0.84)	0.011
Lymphocyte count >3900 cells/µl	4/13	0.99 (0.36 - 2.72)	0.984
Platelets count 1500 - 4000 cells/µl	78/590	1.21 (0.63 - 2.32)	0.573
Platelets count >4000 cells/µl	3/10	2.36 (0.73 - 7.61)	0.151
PLR of 71.38 - 212.3	76/542	2.30 (0.87 - 6.10)	0.095
PLR of > 212.3	9/70	2.31 (0.76 - 7.06)	0.141
White cell count of 4000 - 11000 cells/µl	72/598	0.70 (0.41 - 1.18)	0.181
White cell count of > 11000 cells/µl	5/11	2.01 (0.84 - 4.81)	0.117
MCV 80 - 100 fl	71/491	1.44 (0.88 - 2.34)	0.148
MCV ≥100 fl	1/12	0.79 (0.11 - 5.53)	0.815

IU/L=international units per litre, mEq/dL=milliequivalent per deciliter, cells/µl=cells pre microlitre, fl=femtoliter, mmHg=millimeters of mercury, mm=millimetre.

### Prediction models for preterm birth

All variables with unadjusted relationship <0.2 were included in the models, and later removed stepwise, to leave only those combinations with the least number of variables with the higher AUC. Four models were developed from maternal history, sonographic findings, and laboratory tests. When maternal history and sonographic findings were combined, the sonographic findings became statistically non-significant. Without obstetric ultrasound or laboratory tests (model 1), the predictors of preterm birth were; personal history of preeclampsia, previous history of preterm birth, diastolic hypertension, and multiple pregnancies (Table 3).

**Table 3:** Models for preterm birth derived from maternal socio-demographic characteristics, sonographic findings and laboratory parameters

Variable	OR (95% CI)	p-value
Model 1: Maternal history, physical findings		

History of previous preterm birth	2.10 (1.05 - 3.95)	0.027
Personal history of preeclampsia	11.94 (3.33 - 48.08)	0.000
Diastolic pressure ≥ 90mmHg	3.26 (1.12 - 8.37)	0.019
Multiple pregnancies	13.73 (5.08 - 39.11)	0.000
Intercept	0.10 (0.07 - 0.12)	0.000

Model 2: Maternal sonographic findings		

Unilateral end diastolic notch	0.76 (0.37 - 1.43)	0.418
Bilateral end diastolic notch	1.95 (1.05 - 3.46)	0.027
Intercept	0.13 (0.10 - 0.16)	0.000

Model 3: Maternal laboratory tests		

Serum ALP < 98 IU/L	2.33 (0.82 - 5.72)	0.082
White cell count of 4000 - 11000 cells/µl	0.91 (0.46 - 1.92)	0.798
White cell count of > 11000 cells/µl	3.90 (0.88 - 16.10)	0.063
PLR of 71.38 - 212.3	6.94 (1.84 - 49.3)	0.016
PLR of > 212.3	4.56 (0.92 - 36.86)	0.094
Lymphocyte count 900 - 3900 cells/µl	0.35 (0.14 - 0.92)	0.029
Lymphocyte count >3900 cells/µl	2.29 (0.30 - 21.90)	0.432
Serum urea of 11 - 45 IU/L (10th - 90th percentile)	2.65 (1.04 - 8.99)	0.069
Serum urea of <11 IU/L (<10th percentile)	2.23 (0.55 - 9.62)	0.258
Intercept	0.02 (0.002 -0.15)	0.000

Model 4: Maternal history and laboratory tests		

History of previous preterm birth	2.25 (1.11 - 4.32)	0.019
Personal history of preeclampsia	10.11 (2.68 - 42.07)	0.001
Diastolic pressure ≥ 90mmHg	3.94 (1.34 - 10.39)	0.008
Multiple pregnancies	14.17 (5.09 - 41.72)	0.000
Serum ALP < 98 IU/L	2.35 (0.78 - 6.07)	0.098
White cell count of 4000 - 11000 cells/µl	0.63 (0.33 - 1.25)	0.165
White cell count of > 11000 cells/µl	4.02 (0.92 - 16.09)	0.053
PLR of 71.38 - 212.3	3.78 (1.33 - 14.66)	0.027
PLR of > 212.3	4.07 (1.13 - 18.06)	0.042
Serum urea of 11 - 45 IU/L (10th - 90th percentile)	2.54 (0.96 - 8.97)	0.095
Serum urea of <11 IU/L (<10th percentile)	1.80 (0.39 - 8.52)	0.440
Intercept	0.02 (0.002 - 0.08)	0.000

IU/L=international units per litre, mEq/dL=milliequivalent per deciliter, cells/µl=cells pre microlitre, fl=femtoliter, mmHg=millimeters of mercury, mm=millimetre.

### Evaluation of the models for Prediction of preterm birth

The models were evaluated for AUC, with accuracy, sensitivity, and specificity. The models had a 56.6% – 69.5% area under the curve (AUC), with 52.2% - 62.4% accuracy, and sensitivities of 59.6% - 89.3%. Their specificities of 20.5% - 47.1% was low. Details are in Table 4.

**Table 4:** Model performance evaluation using K-fold cross-validation

Model	Accuracy %	Sensitivity %	Specificity %	AUC %
Model 1 (maternal history)	61.6	87.3	35.0	62.1
Model 2 (Doppler indices)	55.6	89.3	20.5	56.6
Model 3 (Laboratory tests)	52.2	59.6	44.5	62.8
Model 4 (History and Lab tests)	62.4	77.2	47.1	69.5

Without Doppler ultrasound or laboratory tests, the model can detect 87% of those mothers destined to deliver preterm with over 60% accuracy and AUC. This model may be used for screening for preterm birth in prenatal clinics, to aid early referral to higher-level hospitals.

## Discussion

In this study, the development and validation of second-trimester multivariable prediction models for preterm birth at St. Mary's Hospital Lacor, Northern Uganda, was done. Three in every twenty-five mothers (11.6%) got preterm birth. The incidence of preterm birth is 15.7% in Ghana^24^, 6.1% in China^25^, 9.3% in Nepal^26^, and 7.4% in the United Kingdom^27^. That perhaps confirms that women of sub-Saharan Africa are more at risk of preterm birth^28^.

Without uterine artery Doppler ultrasound, or laboratory tests, the predictors of preterm birth were; maternal history of preeclampsia, previous history of preterm birth, diastolic hypertension, and multiple pregnancies. That predicted preterm birth with 66% accuracy of those destined to get preterm birth. Without obstetric ultrasound or laboratory tests, the predictors of preterm birth were personal history of preeclampsia, previous history of preterm birth, diastolic hypertension, and multiple pregnancies. Predictors of preterm birth in Ethiopia were; lack of antenatal care visits, having 1–2 antenatal care visits, history of the previous preterm, short inter-pregnancy interval, having reproductive tact infections, history of abortion, urinary tract infection and hypertensive disorders in pregnancy^29^,^30^. Attending at least secondary education and antenatal care was protective^30^. However, that was a cross-sectional study, and may not reflect the early trimester antenatal characteristics that may be used to predict preterm birth.

The addition of laboratory tests to the maternal history and physical findings slightly improved the model. Bilateral end-diastolic notch was the only statistically significant predictor of preterm birth, with 89.3% sensitivity, 20.5% specificity and 56.6% AUC, when uterine artery Doppler indices were used. However, when combined with a maternal history and laboratory tests, it became statistically non-significant. Therefore, maternal history and physical examination with laboratory tests gave an overall 69.5% AUC, with 62.2% accuracy, 77.2% sensitivity, and 47.1% specificity, for predicting preterm birth.

Our models had AUC ranging from 56.6% to 69.5%, comparable to the systematic review by Meertens et al.^9^, where most models for predicting preterm birth had an AUC of 54% - 70% for both development and validation.

Despite not being statistically significant in our final model, a bilateral end-diastolic notch signifies reduced placental site perfusion, which may translate into placental insufficiency^31^. In Australia, it was found that a bilateral end-diastolic notch would predict preterm birth by 31.4% sensitivity, and 58% AUC^32^. Predicting preterm birth and referral to specialized centres is one of the options for reducing perinatal morbidity and mortality^3^-^5^.

## Strengths of the study

This was a baseline study in Northern Uganda, to find predictors of preterm birth. Due to the low sample size of 774 (for analysis in RStudio), we used all the participants to develop the model. All the participants contributed to the development of the original model. The study predicted preterm birth, with relatively high sensitivity, and AUC, compared to other studies.

## Limitations of the study

There was a high number of mothers lost to follow-up. The validation dataset was generated by over-sampling (many times) those with preterm birth, and under-sampling (a few times) those with term births (ROSE technique in RStudio^21^, ^22^. The result was a dataset with a near-equal number of preterm and term births. The limitation is that some characteristics were over or under-sampled in the validation dataset, which may not represent any average population.

## Conclusion

In this paper, second-trimester predictors of preterm birth were; personal history of preeclampsia, multiple pregnancies, diastolic hypertension, and history of preterm birth. These predicted preterm birth by over two-thirds. However, adding uterine artery Doppler sonography or laboratory blood tests did not significantly improve the prediction models.

## Implications

The models can be used for routine screening for preterm birth in prenatal clinics, to collect more data for its validation. Future research should be undertaken to validate the above models in prenatal clinics in other regions, to ensure generalizability. Policy makers could also interest the funders in incorporating screening for preterm birth in prenatal clinics.
